# Prevalence of musculo skeletal pain among left handed Indian students

**DOI:** 10.6026/9732063002001218

**Published:** 2024-10-31

**Authors:** Alok Desai, Sriniwasan Anandh, Dhirajkumar Mane

**Affiliations:** 1 Krishna College of Physiotherapy, Krishna Vishwa Vidyapeeth (DU), Karad, Malkapur, Karad, Maharashtra - 415539, India

**Keywords:** Musculoskeletal pain, left-handed people (LHP), Nordic Pain Questionnaire (NPQ), quality, right-handed people (RHP)

## Abstract

There are a very small number of left-handed people (LHP) and it is common that most of the equipment in academic institutions starting from benches and laboratory tools to doors and windows are manufactured or modified to be used by right-handed people (RHP) making it difficult to use them by LH ones. Therefore, it is of interest to understand the actual level of pain & its quality in LH individuals caused by the utilization of equipment made for RHP. A total of 50 subjects were asked to fill out the Nordic Pain Questionnaire (NPQ). We found that, the significance of musculoskeletal pain (MS-P) in left-handed individuals is 92%. Thus, the prevalence of MS-P in left-handed students is significant with 92% frequency.

## Background:

A study has shown that, the dominant side of a person is not just the working hand but it also indicates a lot about the MS-P as well as the neurological system of the person. The development of an individual with left-side dominance (LSD) is significantly different than right-side dominant (RSD) individuals in religious countries like India [1]. They have to face a different set of physical, mental as well as social challenges than the rest of the population. This study is mainly focused on musculoskeletal pain MS-P among students with LH-D [1]. A study has shown that MS-D, which includes a number of illnesses that cause pain in bones, joints, muscles, or surrounding tissues, is a recognized outcome of repeated strain, overuse and work-related musculoskeletal disorders [MS-D] [2]. Research indicates that pain may be acute or chronic, localized or diffuse [2, 3]. Additional examples encompass neuropathies, myalgia, stress fractures, as well as tendonitis and tendinosis. A study has concluded that acute MSP is characterized by the sensation of pain localized within a specific region of the body, typically attributed to structures such as muscles, ligaments, bones, or joints in that area. This type of pain is classified based on the affected region, with common examples including back pain, neck pain, shoulder pain, elbow pain, buttock pain, hip pain, knee pain and ankle pain [3]. Research indicates that when musculoskeletal pain persists for more than three months, it is classified as chronic pain [2, 3]. Consequently, duration of three months is recognized as the typical timeframe for the healing process to occur [3]. MSP conditions are common in the work environment and are often connected with mm ergonomic [PE] conditions. PE conditions occur when the work environment is incompatible with the workers' bodies or abilities to continue working. Such conditions may result in discomfort, weariness, pain and subsequent injury [4]. Factors contributing to the discord between the workplace and the employee are termed PE work hazards [WH] [5]. Young adults' lives are substantially impacted by the trouble with persistent MSP, which is particularly important [6]. Therefore, it is of interest to evaluate the prevalence of MSP among LH undergraduate students (UG-S).

## Methods:

The current self-designed electronic questionnaire based study was conducted at KIMS, Karad in 50 LHS to obtain personal and PE details, standardized Nordic questionnaire. Patients were asked to fill out a Google Form which included all the criteria as well as the outcome and only those who had marked the area of pain were required to answer the questions followed by their area of pain; those without any pain did not need to answer any further questions. Each section is followed by 4 sets of yes-no type of questions as mentioned below:-

[1] Have you at any time in the last 12 months had trouble in the given region?

[2] During the last 12 months have you been prevented from carrying out normal activities in the given region?

[3] During the last 12 months have you seen a physician for this condition?

[4] During the last 7 days have you had trouble in the given region?

## Inclusion criteria:

[1] Patients should be LH UG-S.

[2] Age should be between18 to 25 years

## Exclusion criteria:

Those who do not acquire LH due to medical conditions like trauma/polio/paralysis *etc.*

## Statistical analysis:

Significance of the presence of pain was considered according to no. of individuals with pain out of the total sample size in the given region. Furthermore, the quantity of pain in a given region was considered significant if 2 or more questions out of 4 were answered 'Yes'.

## Results:

Table 1 shows that, from the 50 responses collected, 17 participants reported having pain in the neck out of which 12 had a significant intensity of pain, 13 participants reported having pain in the upper back of which 09 had a significant intensity of pain, 11 participants had pain in the shoulders out of whom 07 had a significant intensity of pain, 07 participants reported pain in elbows which 04 had a significant intensity of pain, 04 participants had pain in wrists/hands out of which 01 had a significant intensity of pain, 08 participants had pain in lower back out of which 06 had a significant intensity of pain, other than that 03 individuals had pain hips/thighs but no one had a significant intensity of pain. No participants reported pain in the region of the knees and ankles/feet. A total of 04 participants reported no pain in any region. (Figure 1 see PDF) shows that, the above data suggests that a higher percentage of participants have significant pain around the neck, shoulder and upper back region than lower limbs. A majority of participants (92%) reported having some type of MSP around different parts of the body. While only 8% of the participants reported having no pain.

## Discussion:

The problems these individuals face are not mainly due to the difference in their dominance or their lower population; it is mainly due to the ignorance by the society as well as the individuals themselves. Thus, the goal should be to first address the underlying societal and social causes followed by issuing the appropriate equipment to the students and finally to alter their lifestyle and inculcate ergonomic training. Researchers in another study compared a questionnaire they had developed to measure laterality in orthopedic surgery to a pre-existing scoring system. The validated Waterloo Handedness Questionnaire (WHQ) and the self-developed Orthopaedic Handedness Questionnaire (OHQ) were compared among 62 orthopedic surgeons who had been surveyed [2]. Studies show that compared to LHD surgeons, RHD surgeons had an average WHQ score of 30.2% and an OHQ of 9.4% based on age [6, 2]. This indicates a considerable duration of engagement with the non-dominant hand. However, it does not inherently guarantee adequate or effective dexterity applicable in the surgical environment. Therefore, it may be necessary to implement training for the non-dominant side [2]. Subsequently, another study determined that 15% of orthopaedic surgeons and trainees who participated in the survey were LHD [3]. LHD respondents reported significantly higher rates of ambidexterity in both scalpel/cautery usage and suturing as compared to RHD respondents [2]. As a result, studies indicated that pairing LHD residents with LHD faculty surgeons early in their training may be beneficial in terms of mentorship and insight into performing left-handed surgical procedures [5, 6]. A study also showed that MS-P is significantly linked with sedentary postures and inadequate engagement in structured physical exercise among health science UG. In terms of MSP prevalence, the logistic model was able to accurately classify 94.1% of instances while also explaining 23.6% of the variance (χ^2^=13.73, p=0.03) [3]. A study showed that left-handed medical students reported more ambidexterity and a negative influence of handedness on training. These findings provide a current snapshot of hand preference among medical students, as well as a case for upgrading and adjusting surgical training for left-handed people [1]. A study indicated that male students were better than female students at differentiating between right and left, while aspiring surgeons outperformed aspiring general practitioners or medical professionals. Students struggled more with the front view than the rear view [2].

## Conclusion:

Data shows that 92% of left hand (LH) individuals suffer from musculo-skeletal pain in different regions of their body which makes a serious consideration about changing or modifying the environment surrounding LH individuals according to their PE needs. Further studies should be done to get a thorough assessment of musculo-skeletal pain in the upper region of the body (*i.e.* neck, upper back, shoulders) in left-handed individuals.

## Figures and Tables

**Table 1:** Distribution of individuals according to significance of MCP

**Region of Pain**	**No. of individuals with Significant pain**	**No. of individuals without significant pain**	**Total no. of individuals (N=50)**	**Percentage (%)**
Neck	12	5	17	34%
Upper Back	9	4	13	26%
Shoulders	7	4	11	22%
Elbows	4	3	7	14%
Wrists/Hands	1	3	4	8%
Lower Back	6	2	8	16%
Hips/Thighs	0	3	3	6%
Knees	0	0	0	0%
Ankles/Feet	0	0	0	0%
No Pain	-	-	4	8%
